# Generative Adversarial Networks–Enabled Human–Artificial Intelligence Collaborative Applications for Creative and Design Industries: A Systematic Review of Current Approaches and Trends

**DOI:** 10.3389/frai.2021.604234

**Published:** 2021-04-28

**Authors:** Rowan T. Hughes, Liming Zhu, Tomasz Bednarz

**Affiliations:** ^1^ Expanded Perception and Interaction Centre (EPICentre), Faculty of Art and Design, University of New South Wales, Sydney, NSW, Australia; ^2^ CSIRO Data61, Australian Technology Park, Eveleigh, NSW, Astralia

**Keywords:** machine learning, artificial intelligence, generative adversarial networks, GANs, human-in-the-loop, human–artificial intelligence collaboration

## Abstract

The future of work and workplace is very much in flux. A vast amount has been written about artificial intelligence (AI) and its impact on work, with much of it focused on automation and its impact in terms of potential job losses. This review will address one area where AI is being added to creative and design practitioners’ toolbox to enhance their creativity, productivity, and design horizons. A designer’s primary purpose is to create, or generate, the most optimal artifact or prototype, given a set of constraints. We have seen AI encroaching into this space with the advent of generative networks and generative adversarial networks (GANs) in particular. This area has become one of the most active research fields in machine learning over the past number of years, and a number of these techniques, particularly those around plausible image generation, have garnered considerable media attention. We will look beyond automatic techniques and solutions and see how GANs are being incorporated into user pipelines for design practitioners. A systematic review of publications indexed on ScienceDirect, SpringerLink, Web of Science, Scopus, IEEExplore, and ACM DigitalLibrary was conducted from 2015 to 2020. Results are reported according to PRISMA statement. From 317 search results, 34 studies (including two snowball sampled) are reviewed, highlighting key trends in this area. The studies’ limitations are presented, particularly a lack of user studies and the prevalence of toy-examples or implementations that are unlikely to scale. Areas for future study are also identified.

## 1 Introduction

The emergence of artificial intelligence (AI) and machine learning (ML) as a crucial tool in the creative industries software toolbox has been staggering in scale. It is also one of the most active research areas in computer science (Murphy, 2012). Recent progress in generative deep learning (DL) techniques has led to a dearth of new solutions in the fields of computer graphics, vision, and user-aided design (Pan et al., 2019). ML applications that directly interface with everyday users are increasingly pervasive. However, these applications are still largely entirely designed and deployed by ML engineers. Data collection, feature selection, preprocessing, model development, parameter tuning, and final assessment of the resulting model’s quality are all made without consulting with end-users on how they will interact with the resulting system. Typically, this has led to systems where the end-user involvement consists of little more than providing some input and hoping for a good result. In this survey, we look at research where end-users’, specifically design practitioners’, involvement is deeper and collaborative in nature. That is, systems that function as design support tools rather than simple automatic synthesis tools.

One of the main challenges facing DL today is its “black-box” nature. Data are fed to a trained neural network, which then outputs a classification, decision, action, sample, etc. Despite recent advances in the field of explainable artificial intelligence (XAI) (Biran and Cotton, 2017), these algorithms’ inner workings often remain mysterious to the user and even to the model’s engineers. While the architecture and mathematics involved are well-defined, interpreting what is happening in the neural network’s inner state remains a very challenging problem (Zeiler and Fergus, 2013). This opaque nature can also lead to a fundamental mistrust between end-users and the systems with which they are interacting. The emergence of the family of generative models has created another potential avenue for the erosion of trust, with much-publicized examples such as systems to hide from facial detection systems (Mirjalili et al., 2017; Johnson, 2020) or the generation of highly realistic fake images (Zhu et al., 2017a) having drawn mixed public reaction. Exploring these issues is an essential and complex research area. One way to address trust is to give the user real-time or interactive feedback, allowing them to visually explore and develop a relationship with the underlying system. Finally, there is the ethical perspective (Whittle, 2019; Fjeld et al., 2020). To minimize potential harm to society, there needs to be a strong commitment from both government and society to provide oversight and regulation concerning how and where AI systems are used.

**FIGURE 1:**
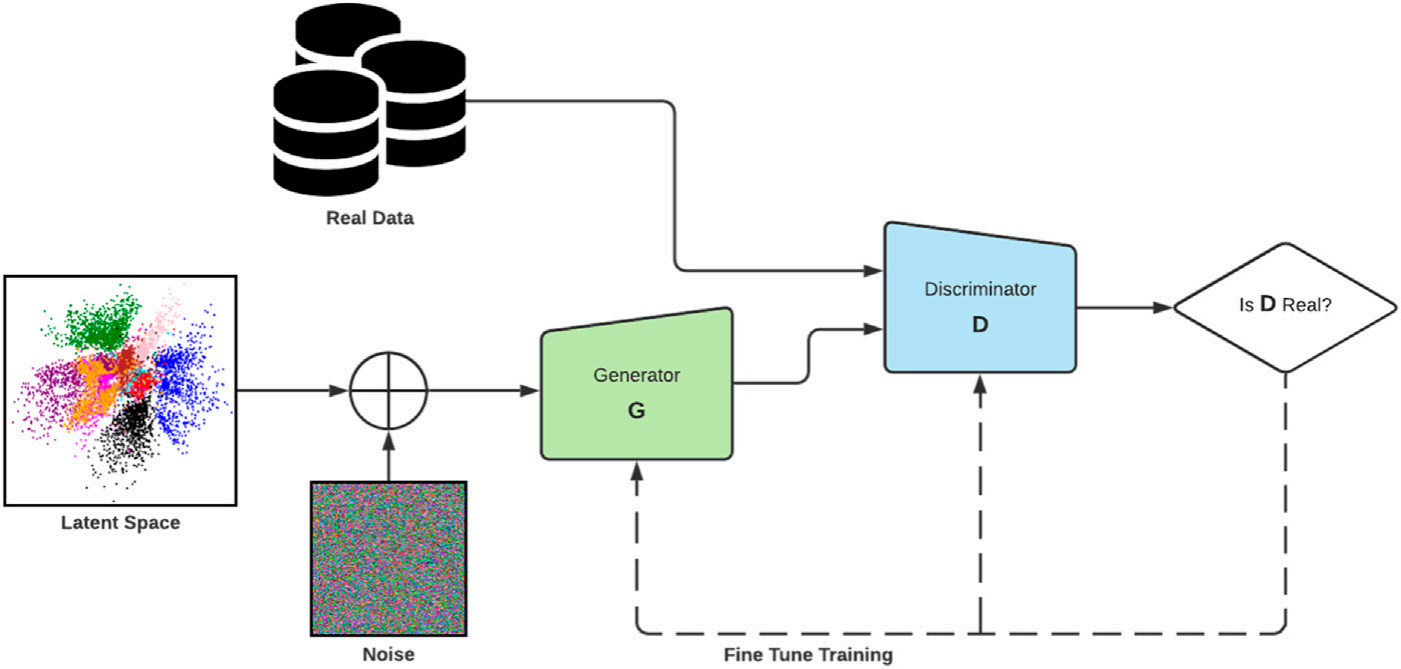
The original GAN architecture as described by Goodfellow et al. (2014).

One of the most decisive steps forward in DL synthesis has been the development of the family of algorithms known as generative adversarial networks (GANs). First proposed by Goodfellow et al. (2014) in 2014, GANs are a type of generative model with a specific architecture in which two networks, a generator and a discriminator, compete with one another to produce increasingly plausible generated samples (Figure 1 shows the original architecture). In practice, a GAN is not dissimilar to any other convolutional neural network (CNN). The discriminator’s core role in a GAN is similar to an image classifier, and the generator also operates similarly to other CNNs, just operating in reverse. GANs have several advantages over other members of the deep generative model family of algorithms. They produce higher quality output (Goodfellow, 2017) than other models. When compared with variational autoencoder (VAE), the images produced by GANs tend to be far sharper and realistic (Goodfellow, 2017). Auto-regressive models (van den Oord et al., 2016) have a very simple and stable training process, but they are relatively inefficient during sampling and do not easily provide simple low-dimensional codes for images. The GAN framework is flexible and can train any type of generator network. Other models have constraints for the generator (e.g., the output layer of the generator is Gaussian (Kodali et al., 2017)). There is no restriction on the latent variable’s size. These advantages have led to GANs leading performance in generating synthetic data, especially image data (Wang et al., 2019).

An important step toward integrating GANs into design tools was developing methods to add a level of control over the generated outputs. Conditional GANs (Mirza and Osindero, 2014; Lee and Seok, 2017) allow the user to add additional input values to the generator and discriminator for categorical image generation. The InfoGAN (Chen et al., 2016) algorithm can extract latent features in an unsupervised manner by introducing a latent code, which is fed as an additional input to the generator. The latent code can then capture the generated images’ structure by adding an additional regularization term to the loss function of GAN between the latent code and the generated image. Research into user control over generative networks is a very active area, but still in early development (Carter and Nielsen, 2017) but maturing rapidly (Pan et al., 2019).

A full breakdown of the current state of the art concerning GANs is outside this study’s scope. There have, however, been many excellent recent surveys on the state of the art (Alqahtani et al., 2019; Hong et al., 2019; Pan et al., 2019; Khursheed et al., 2020), performance (Kurach et al., 2018), advances in image synthesis (Wu et al., 2017; Wang et al., 2019), and approaches to improving stability (Wiatrak et al., 2019). These reviews showcase the prevalence of GANs in research and indicate it as a growing area of importance. While there have been individual case studies into interactive systems that look at collaborative design with generative models (Kato et al., 2019; Noyman and Larson, 2020), there has not been a systematic review looking at the area more broadly.

In the next section, we will qualify the reasoning for selecting human–AI collaborative design with GANs as an area for further investigation and describe the work’s guiding research questions. Following this, the systematic literature review methodology will be described, and the results of the review will be presented.

## 2 Motivation

The families of generative models can be broadly categorized into two distinct categories: explicit density and implicit density models. Explicit density models are those models that assume some kind of prior distribution about the data. Prevalent examples of these approaches are those based around recurrent neural networks (RNNs) (van den Oord et al., 2016), autoencoders (VAEs) (Dayan et al., 1995), and their variants (Kingma and Welling, 2013; Oord et al., 2016; Higgins et al., 2017). These approaches have produced excellent results and are widely studied (Zhai et al., 2018). However, they do have some drawbacks that limit their current adoption in creative tools. Models based around RNNs operate sequentially; therefore, output generation is comparatively slow. Autoencoders do not exhibit this problem but have not been shown to produce the output quality of competing models. GANs are a very prevalent example of an implicit density model, models that do not explicitly define a density function. Despite the drawbacks associated with GANs, such as training stability, they currently exhibit several advantages over competing models. Most importantly, they do not have the performance issues exhibited by RNN-based models while generating best-in-class output quality. For this reason, we focus on research that utilizes GANs and investigate their increasing prevalence as a tool in the process of design.

As mentioned previously, much of the current research around GANs has focused on automatic synthesis (Wang et al., 2019; Khursheed et al., 2020), where end-user interaction with the system is minimal (e.g. image-to-image translation). These systems have also been improving swiftly and are now capable of some impressive results (Wu et al., 2017). Despite this, however, interaction with AI technology as a design tool is still a relatively immature and challenging problem (Dove et al., 2017), so we take a more targeted look at the current research that has a more collaborative approach to human–AI interaction.

### 2.1 Benefits

Generative design has a broader history and scope beyond the ML space (Krish, 2011). Traditionally, these tools have been used by engineers or design experts who input design goals and parameters (e.g., performance, mass, spatial, and cost requirements). From these inputs, these tools explore the solution space, generating design options and alternatives. AI technology has been making inroads in the area^1^ (Kazi et al., 2017), and we are now beginning to see generative models coming into the fold at research and application levels.

**FIGURE 2:**
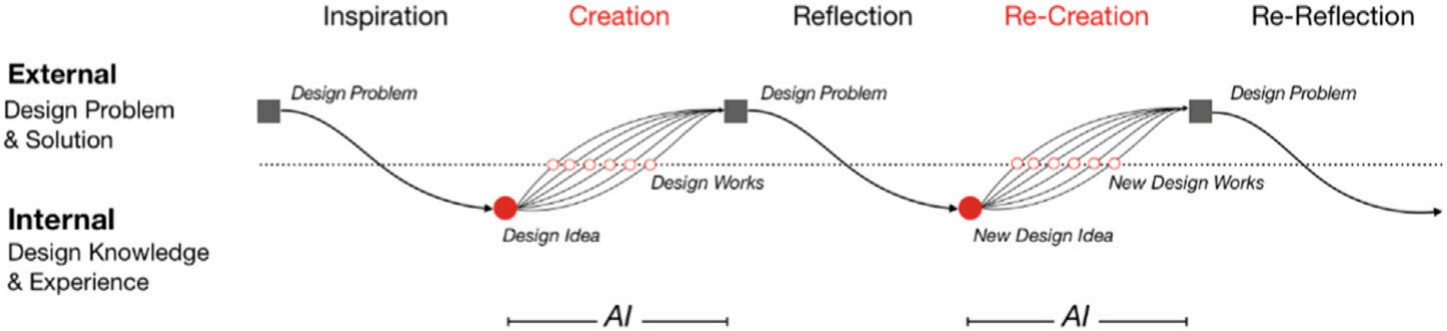
The AI-augmented creative design cycle as described by Zeng et al. (2019). The authors describe how AI can be used to augment the design process by introducing variety to the users’ input, allowing them to quickly expand their solution space. The core creative design cycle of continuous interaction between creation and reflection remains unchanged.

Figure 2 illustrates how Zeng et al. (2019) saw AI being integrated into the design cycle. It shows how the human–AI relationship can be collaborative; in this case, their system generates design variety from user input, which can then be explored by the user and incorporated into the next iteration. Other examples we will discuss include systems that can generate landscape paintings (Sun L. et al., 2019) or terrains (Guérin et al., 2017) from quick sketches, thus allowing users to more efficiently iterate over their designs than if they had to realize their design at each step fully.

ML has been an active research area for a long time, but its adoption as a mainstream technology in the creative/design space is a relatively new phenomenon. Much of the research has been directed into creating systems capable of performing tasks, and while many powerful systems and applications have emerged, the user experience has not kept pace (Dove et al., 2017; Yang, 2018). Interaction design aims to create interfaces that are easy to use, effective, and enjoyable. In designing user interfaces that interact with AI systems, there has been a general lack of focus on the end-user. This review will look at many examples where interaction design and end-user needs have been considered to varying degrees, highlighting good practice, current limitations, and avenues of interest for further research.

### 2.2 Challenges

In the public mind, the use of AI and ML is seen as a new, innovative technology. While this notion has some truth to it (Cearley et al., 2019), it is also true that ML is quite a mature field. ML has been around a long time (Samuel, 1959). There are countless textbooks, academic courses, and online resources dedicated to the topic. With this said, user experience design for ML systems and human–AI collaboration remains relatively rudimentary (Dove et al., 2017; Yang, 2018; Yang et al., 2020). There may be several reasons for this, but one that stands out is simply that ML is a fundamentally more difficult design material. ML systems have a “black-box” quality to them that is fundamentally different from heuristics driven systems. The outputs of these systems are often not easily explained, particularly when errors occur. Therefore, designers have a challenging task designing systems that bridge the ML–human perspective, with deep collaboration with engineers being critical.

The research challenges associated with improving human–AI collaboration in the generative space do not have the easily digestible outcomes associated with the most well-known work in the DL field. To evaluate the work, one asks the question: “Is the technology helping humans think and create in new ways?” rather than whether the technology outperforms previous methods on a well-defined task. This can be a more difficult question to ask.

There is an outstanding question as to whether creativity might be limited by using tools based on GAN architecture. An optimally trained GAN generator should recreate the training distribution and therefore cannot directly generate an image based on new governing principles because such an image would not be similar to anything like it has seen in its training data. Therefore, one must ask if users would be prevented or discouraged from exploring more exciting directions. While GANs show tremendous promise in allowing people to create and explore, this fundamental question remains.

Aside from human–AI interaction challenges, many technical challenges exist despite the powerful results demonstrated by GANs. One issue is mode collapse, which is one of the most common failures in GANs. It occurs when the generator maps multiple distinct inputs to the same output, which means that the generator produces samples with low diversity. There are many proposed solutions (Arjovsky et al., 2017; Kurach et al., 2018) to mitigate the problem, but it remains an area of research. Another problem is training convergence. As the generator improves with training, discriminator performance naturally decreases because it becomes increasingly more difficult to distinguish between real and fake. This progression poses a problem for convergence of the GAN as a whole: The discriminator feedback gets less meaningful over time. If the GAN continues training past the point when the discriminator is giving completely random feedback, then the generator starts to train on junk feedback, and its quality may collapse. Finally, it is worth mentioning that the training of a simple neural network takes some computational effort. There is an added level of effort required in training GANs due to the networks’ dueling nature, requiring both more time and computational horsepower. While these technical challenges are not the central focus of this paper, they represent a significant factor in how the GAN-enabled user interfaces are developed and deployed.

## 3 Methodology

With ML, more specifically GANs, becoming increasingly important for a range of reasons previously described, and work in this area beginning to grow, it is important to take stock of the current approaches to find similarities, themes, and avenues for further research. As such, the guiding research questions for this review are as follows:

• What approaches exist around GAN-enabled human–AI collaborative design tools?
• What are the limitations of studies and approaches around GAN-enabled human–AI collaborative design tools?
• What subareas are understudied in the domain of GAN-enabled human–AI collaborative design tools?

Given these research questions, the following section describes the methodology for searching the extant literature for information to address them.

## 4 Literature Selection Criteria

A systematic literature review was performed using the PRISMA (Shamseer et al., 2015) reporting methodology to examine the current state of the literature. Searches were conducted on the ScienceDirect, SpringerLink, Web of Science, Scopus, IEEExplore, and ACM digital libraries, using the following Boolean search queries:

• (“Generative Adversarial Network” OR “GAN”) AND (“Art Design” OR “Sketch” OR “Computer Art” OR “Artist” OR “Creative Arts” OR “Computer Aided Design”)
• (“Generative Adversarial Network” OR “GAN”) AND (“Architecture” OR “Urban Design” OR “Urban Planning”)
• (“Generative Adversarial Network” OR “GAN”) AND (“Design Process” OR “Computer Aided Design” OR “Human Computer Interaction” OR “Human-AI” OR “Collaboration”)
• (“Machine Learning”) AND (“Art Design” OR “Sketch” OR “Computer Art” OR “Artist” OR “Creative Arts” OR “Computer Aided Design”)
• (“Machine Learning”) AND (“Architecture” OR “Urban Design” OR “Urban Planning”)
• (“Machine Learning”) AND (“Design Process” OR “Computer Aided Design” OR “Human Computer Interaction” OR “Human-AI” OR “Collaboration”)

In addition, articles were restricted using criteria common in systematic reviews in the area of ML. The criteria used were as follows:

• Recent article: articles had to be published within the last 5 years (i.e., since 2015 at the time of writing);
• Relevancy: articles had to be relevant to the topic of AI (articles which spoke about general AI learning from a human psychology perspective were excluded) and future of work (i.e., articles which did not describe approaches or techniques for advancing the future of work were excluded);
• Accessibility: articles needed to be accessible via the portals previously described;
• Singularity: duplicate articles were excluded;
• Full paper: abstracts and other short papers were excluded (extended abstracts were included).

**FIGURE 3:**
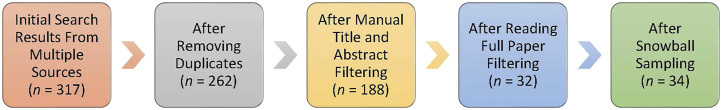
Number of articles included in the review after various stages of filtering.

Figure 3 illustrates the filtering process used to produce the final set of literature. Using the above research parameters, combined with a year filter (≥2014), a total of 317 articles were gathered, which were reduced to 262 after filtering out duplicate results using the JabRef software “Remove Duplicates” feature. The titles and abstracts of these articles were reviewed for relevance to the domain of generative networks and design, of which 188 were deemed relevant using the relevancy measure described above. These articles were then read in full to determine relevance to the domain. The remaining 34 articles after this stage of filtering constitute the primary analysis of this article.

The collapse from 317 to 34 works was due to the search terms’ broad scope. Many of the articles returned outlined automatic methods or algorithms. The criteria for this survey require the method to be user-guided or have iterative user involvement, so these articles were excluded from the final literature. Second, several articles simply mentioned the search terms for describing AI systems generally for the reader. Such passive use of the search terms could not be determined until the full paper was examined.

Additionally, two articles were added to the review, using a snowball sampling technique (Greenhalgh and Peacock, 2005), where if a reviewed article cited a relevant sounding article, it was subsequently assessed, and if deemed relevant, added to the pool of articles for review (14 articles were examined during this stage).

Before discussing the methodologies, the following section explores at a high level the core themes in the 34 articles reviewed, in terms of example domains and scope, to paint a picture of the current state of the research space.

## 5 Summary of Literature

Selected articles were categorized and analyzed based on domain space, publication type, year, user-interface modality, and operation method. A full list of selected articles and values for each of these is provided in the appendix.

### 5.1 Publication Type

The reviewed articles’ largest outlet was conference proceedings (18), with 15 articles published in journals. One extended abstract (Noyman and Larson, 2020) was included due to its scope and critical relevancy.

### 5.2 Year

**FIGURE 4:**
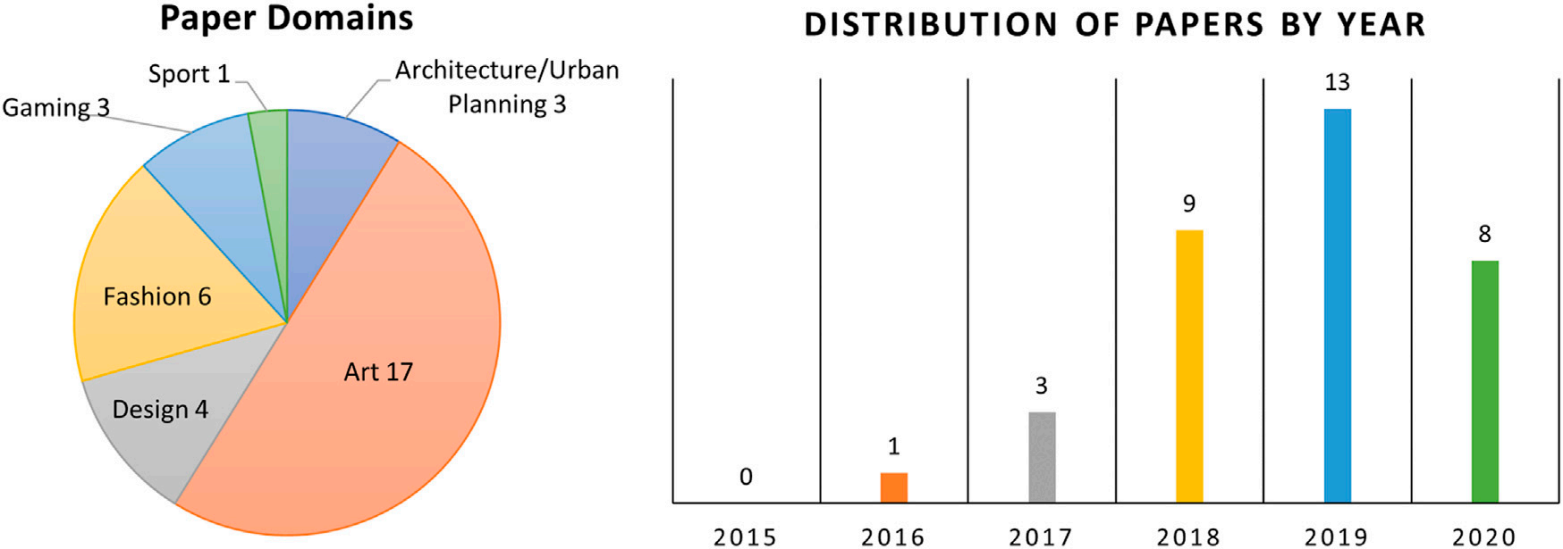
**Left:** Categorization of articles by domain. **Right:** Distribution of surveyed articles by year, indicating an increase of academic interest in this area.

In 2019, 13 articles were published. Eight were published in 2020 (so far), nine in 2018, three in 2017, and one in 2016 (Figure 4). This indicates that research into attempting to incorporate GANs with user-guided design is an area that is young and developing. The slight decrease in articles in 2020 may be due to the current difficulties in performing both experiments and user testing. Given the sudden increase in publications, there is a reasonable amount of cross-over between some research streams. Ideally, these researchers may consolidate their work and progress together, rather than in parallel, into the future.

### 5.3 Domain Space

Articles were categorized based on the featured subject domain(s) they focused on (either in their implementation or theoretical domain). Work could exist across multiple categories. The distribution of articles across the categories is summarized in Figure 4 and expanded upon in this section.

The largest cohort of articles (17 articles) focused primarily on art-design tasks (e.g., generating paintings, and terrains). Six articles are situated in the fashion-design space. An area that was expected to have greater representation was urban planning/design; however, this area was the focus in only three articles reviewed. There were four articles addressed in the graphic design space and three in game design. Finally, one article addressed sports-play design. This categorization helps understand the areas where this research is currently being deployed and aids in identifying areas currently under-served.

### 5.4 Human–Computer Interface Modality

**FIGURE 5:**
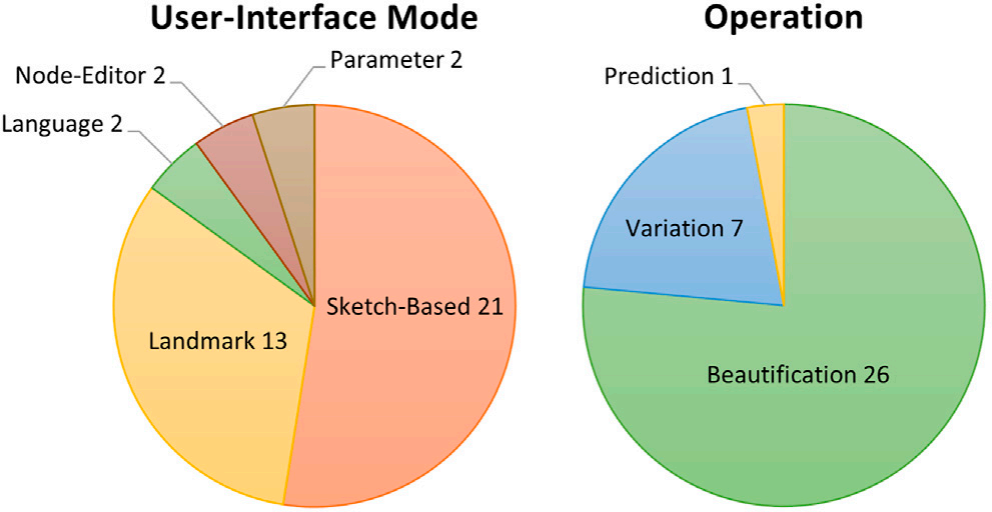
**Left:** Categorization of articles by UI modality. Note that some articles were multifaceted and covered multiple categories. **Right:** Categorization of articles by UI modality. Note that some articles were multifaceted and covered multiple categories.

We are focused on researching how GANs are being integrated into design support tools. One of the critical human–computer interaction (HCI) considerations when creating these tools is how the end-user will communicate or interact with the system. The reviewed articles present various interface modalities, with the most common being sketch-based interfaces (21 articles) and what we choose to call “landmark-based,” which is where an area or point of interest is marked by the user in some manner (12 articles). In addition, two works each featured node-graph, parameter-based, and language-based interaction modalities, respectively. Figure 5 illustrates the breakdown of the UI modalities.

As most articles are situated within the art-design space, it is somewhat unsurprising that most interface modalities were sketch-based. Sketch-based UI systems are familiar, comfortable, and intuitive to use for artists. There is some cross-over between sketch and landmark modalities also, as sketches can be used to provide information, or as commonly referred to as “hints,” to the network to constrain or guide the output. Node-based interfaces are another common feature of modern digital content creation (DCC) tools. This type of interface may become more prevalent in the future.

Natural-language user interfaces (NLUIs) have become a feature of everyday life (Shneiderman et al., 2016). It remains a challenging problem and a highly active research area. Despite this, NLUIs represent a highly intuitive way to communicate with a system. Two of the articles reviewed took this approach.

Disentangled representation learning (Locatello et al., 2019) is an exciting, emerging research topic within the ML space. It is an unsupervised learning approach that seeks to encode meaningful feature representations in the learned space, with each dimension representing a symmetrically invariant feature. In practical terms, this allows for the extraction of parameters that correspond to desirable features that facilitate control over the system. If we take the example of a data set of simple shapes, the approach may allow for the extraction of parameters such as rotation and color. This is not a trivial task within the GAN space, as there is no predefined distribution over which we can exercise control. Two articles adopt current approaches (Chen et al., 2016) to the problem to present users with controllable parameters to aid in the design process.

### 5.5 Method of Operation

In examining the surveyed work, two fundamental modes of operation became apparent: variation and beautification.

Design horizon expansion through variation is not a new paradigm. Many interesting new tools have been coming online to allow designers to explore machine-generated variations. The basic workflow is that a designer provides a design, possibly alongside a specified set of constraints (e.g., variance from example and structural constraints). The machine then generates a selection of design variants. The designer can then examine the variants and select one or adapt their design, taking inspiration from the generated examples. This process can be iterated over until a final desired design is found. Seven articles fall into this category.

The other primary mode of operation was “beatification,” or elaboration based on course user input. This mode is perhaps the most straightforward mode of operation, in that designers provide the system with course level input (e.g., sketches, graphs, and language-based instruction), and the system outputs a more fully realized design (e.g., image, landscape, and game level). This review outlines various examples of this approach, and despite differences in interaction mode, inputs, and so on, the basic principle remains the same. This category represents the largest cohort of works, with 26 articles.

A single outlier, BaketballGAN (Hsieh et al., 2019), operates by generating a predicted simulation result given a user design.

## 6 Discussion

### 6.1 Research Question 1: What Approaches Exist Around Generative Adversarial Networks-Enabled Human–Artificial Intelligence Collaborative Design Tools?

#### 6.1.1 Architecture and Urban Planning

**FIGURE 6:**
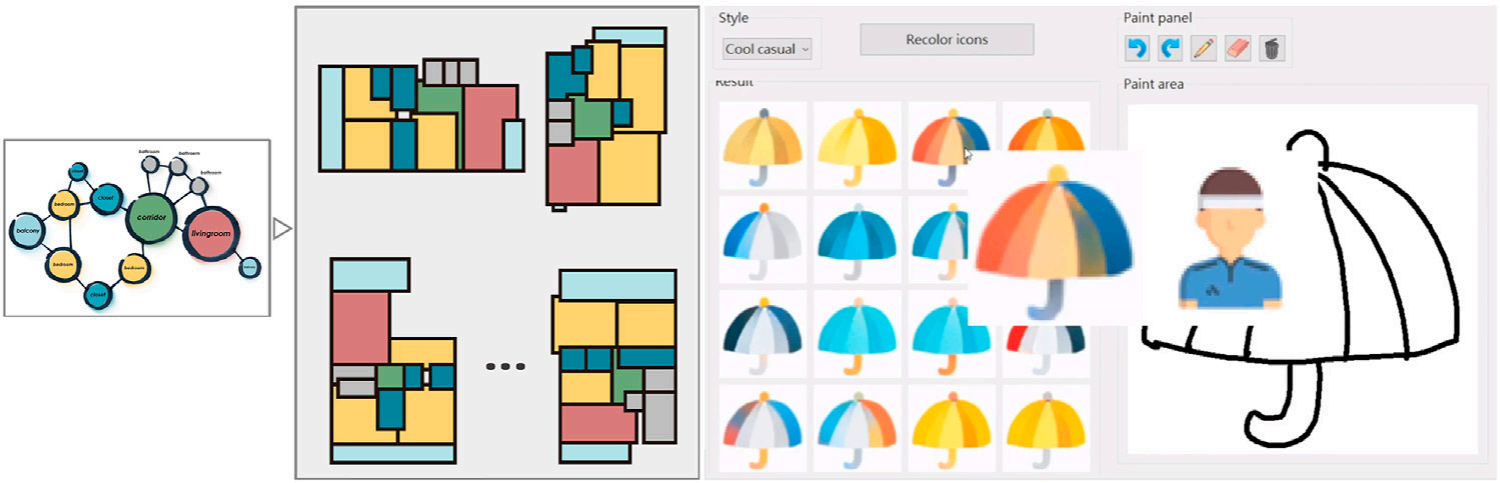
**Left:** The interface for the HouseGAN system (Nauata et al., 2020). The user describes a node-graph, with nodes representing rooms and edges the connections between them. The system outputs a variety of designs based on the graph and the user can then further iterate and design to reach a final floor plan. **Right:** In the work by Sun T.-H. et al. (2019), the authors developed an interface to help designers develop digital icons. The user specifies a sketch and a style they would like to follow. The system then outputs icons based on the input sketch and style in a variety of colors.

Graph editors are a common interface paradigm within the DCC landscape,^2^ ^,^ ^3^ so it is somewhat interesting that the work by Nauata et al. (2020) presented one of only two graph editor interfaces in the reviewed literature. The work describes a framework for a node-graph–based floor plan generation tool. A user constructs a simple graph representing the rough desired layout (Figure 6), with nodes representing rooms of various categories and edges representing adjacency. The method uses the Conv-MPN (convolutional message passing networks) (Zhang et al., 2019) architecture, but here, the graph structure is explicitly passed to the generator. The Conv-MPNs are used to update feature volumes via message passing, which are later up-sampled and propagated to a final CNN network that converts a feature volume into segmentation masks. In this way, the generator generates output that resembles a floor layout, a segmented image with axis-aligned rectangles for each room and corridor. The user can then select preferred outputs and manually adjust them as required. The work notes some limitations to be addressed in future work, such as the current requirement that rooms be rectangular and allowing for further parameterization (i.e., room size and corridor length).

The FrankenGAN (Kelly et al., 2018) framework allows users to generate high-fidelity geometric details and textures for buildings. The name is due to its nature as a patchwork of networks rather than an end-to-end system. By adopting this approach, intermediate regularization steps could be performed, leading to higher quality results. In addition, it offers users the chance to interact with the system at several stages. A user initially provides a coarse building shape, and the geometry generation network then adds high-fidelity details, such as doorways, windows, and sills. At this stage, the user can edit the generated geometry through a sketch-based system before passing new geometry to the texture generation network. The user can then specify the desired style for the resulting texture as well as variance parameters. The authors present some impressive results across a wide range of building styles, and a perceptual study indicated that their system produced significantly better results than competing models.

**FIGURE 7:**
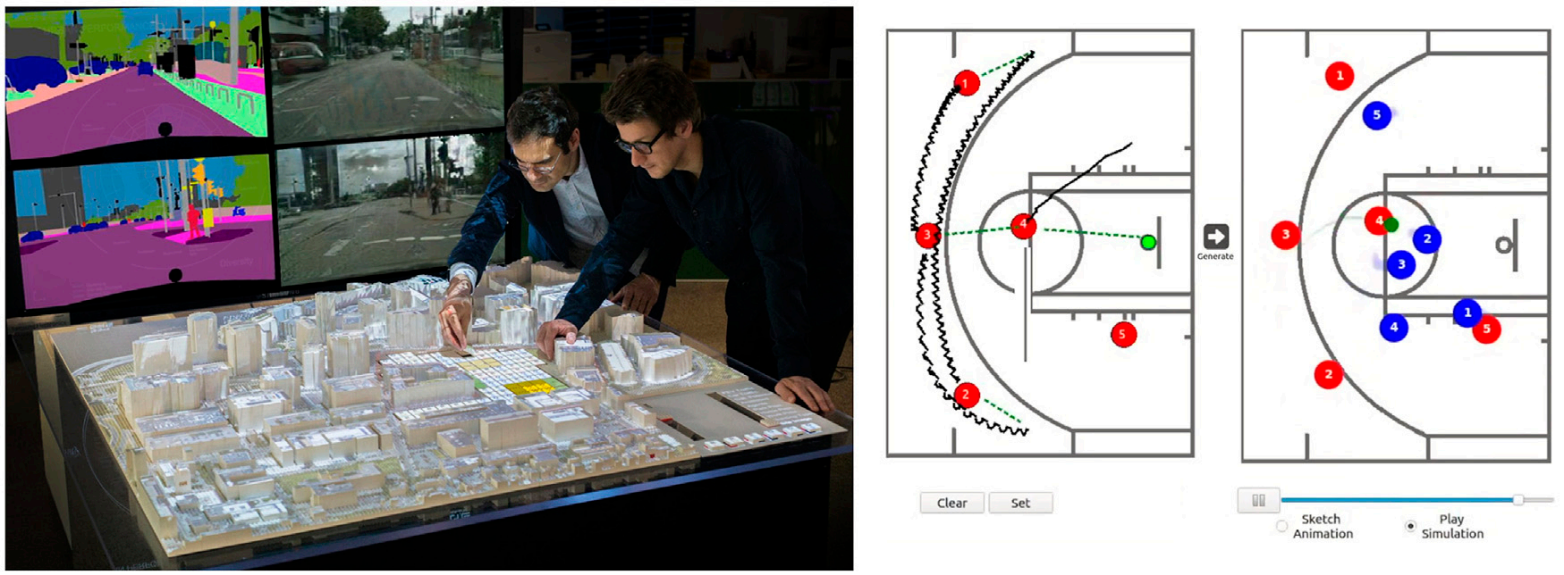
**Left:** The TUI interface for the DeepScope system. Here, the designers move about physical objects representing urban tile classes and can view the cityscape emerge in real time. **Right:** The user interface for BasketballGAN looks much like a traditional basketball clipboard sheet.

The DeepScope project (Noyman and Larson, 2020) presents a real-time, generative platform for immersive urban-design visualization. In this work, the authors used a tangible user interface (TUI) to allow designers to iterate over urban designs quickly and observe generated images of the emergent street scene in real time (Figure 7: top left). The TUI takes the form of a tabletop with a grid layout, with physical cell blocks of varying colors representing different classes of tile (street, green space, and construction) and an observer (a standard LEGO figurine), from whose perspective the final street scene is rendered. A scanner overlooks the tabletop and relays changes being made to the grid-cell layout, which updates the system’s internal virtual 3D layout. This 3D environment is procedurally decorated with cityscape elements (e.g., lamppost and vegetation) and then passed to the DC-GAN, which generates a street scene from the observer’s perspective. The system was designed with intuitiveness as a design principle, allowing experts and nonprofessionals to experiment collaboratively with urban design scenarios with real-time feedback. The platform can augment the early stages of cityscape design with vivid street-view visuals. Unlike traditional CAD tools, the complexity of creating a 3D urban scene is carried out by the pretrained neural network. Finally, the lack of high-resolution visual fidelity, currently a drawback with GAN output, allows designers and regulators to focus on the overall “Image of the City” instead of undecided details.

#### 6.1.2 Graphic Design

Sun T.-H. et al. (2019) presented a system that aids designers in the creation of digital icons. The authors trained a dual-conditional GAN (Yi et al., 2017) on a large database of icons. In this case, rather than training a discriminator to recognize whether an icon is man-made or machine-generated, two discriminators determine whether paired images are similar in structure and color style, respectively. With this system, humans and machines cooperate to explore creative designs, with human designers sketching contours to specify the structure of an icon, then the system colorizing the contours according to the color conditions. To improve usability and to not overwhelm the user with all possible varieties, the user is asked to specify a “style.” The system then randomly selects a selection of icons labeled with that style which are fed to the network as the color condition. Even giving for the fact that output are relatively simple icons, the results of this method are quite impressive and a subjective evaluation study conducted by authors confirmed that their method performed best among a selection of other methods representing the state of the art.

Content layout is a core skill of graphic designers, being a key component in guiding attention, esthetics, etc. Zheng et al. () presented a system for generating user-guided high-quality magazine layouts. The network was trained on a large data set of fine-grained semantic magazine layout annotations with associated keyword-based summaries of textual content. Users can exercise control over the layout generation process by roughly sketching out elements on the page to indicate approximate positions and sizes of individual elements.

The work of (Zeng et al., 2019) investigated whether AI can be used to augment design creativity. They based their approach on fundamental design principles (Dorst and Cross, 2001; Preece et al., 2015) and adapted the existing design cycle to incorporate AI tools (Figure 2). Addressing a particularly difficult design problem, that of typeface design for Chinese characters, the authors noted the historically stylistically broad yet unified nature of Chinese character design. Modern Chinese character fonts do not exhibit this level of variation, and so the authors attempt to use AI to augment typeface designers’ creative abilities. The network was trained on a selected number of standardized Chinese typefaces. The final model was then used to generate many typefaces, and the designers examined the generated fonts to find ones that matched their desired features. Input fonts that adversely affected the resulting sets were then removed, and the network was retrained. This cycle was repeated until the designer’s criteria were met. The study shows how the design cycle is not fundamentally altered, but simply augmented. Design professionals are still responsible for putting forward questions, formulating rules, and providing the starting point. The AI can then take over some responsibility, to generate a more diverse set of typeface forms than would be feasibly possible by a design team. The study also demonstrates how this collaboration can continue indefinitely to meet design goals.

Chen et al. (2019) presented a system to aid design ideation. The framework consists of two separate networks: a semantic ideation network and a visual concepts synthesis network. In the initial design session, the users interact with a visual semantic network graph (the network is based on ConceptNet, with a filter to increase concept diversity). The users can choose how far they would like to venture, conceptually, from the initial idea and also view and filter the resulting network (i.e., in one example, a user starts with the concept “spoon,” steps forward, and lands on “straw” via “soup”: the participant then combined the two ideas to develop a spoon that incorporated a straw into the handle). In the second phase, the system uses a GAN to generate novel images that attempt to synthesize a set of visual concepts. A case study was performed, with participants developing some interesting results, combining concepts together (e.g., one design team blended a spoon with branching leaves, each of which had a vessel for condiments shaped like seeds) in unusual and interesting ways.

#### 6.1.3 Game Design

In the field of content creation for game design, Volz et al. (2018) presented a controllable level creator for the ever-popular Mario Brothers video game. Many, including modern level descriptions for tile-based 2D platformers, boil down to simple text files. In this work, a DC-GAN was trained on a large set of text-based Mario Brother levels. To provide designers a level of control over the final levels, the space of levels encoded by the GAN is further searched using the covariance matrix adaptation evolutionary strategy (CMA-ES) (Hansen et al., 2003). This algorithm makes it easy to specify vectors that correspond to various desirable features, such as the number of enemies and bonuses. One issue that arose was that some of the generated levels were unsolvable and unreachable by the player, given the control restrictions. This was solved through a simulation approach. An AI agent ran through each of the generated levels to identify those levels that did not have a solution. In this way, level elements with undesirable features were eliminated. The authors note the fast speed of level generation and suggest that the levels could be generated on the fly, dynamically adapting to play-style, difficulty, or designer input.

**FIGURE 8:**
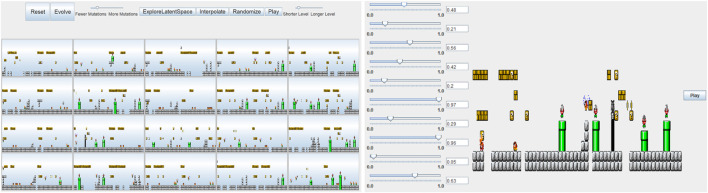
The level design interface designed by Schrum et al. (2020). **Left:** The user can select any number of level segments with desired features and evolve new segments. **Right:** The user can further refine segments through direct manipulation of latent vector values.

Schrum et al. (2020) extended this work with a focus on providing a set of design tools that would give level designers a greater level of control over the finished levels. They again used a GAN to generate a latent space, from where level segments can be drawn. The designer can explore a series of level segments, highlight segments they like and then apply a latent variable evolution algorithm that presents them with the selected segments and their offspring for further iteration. The designer can also select an individual-level segment and alter the individual latent vector values, allowing further control. The designer can also select two segments and interpolate between them by walking the line in high-dimensional space between the two latent vectors (Figure 8). The authors then performed a user study, with 22 participants, to evaluate their system. Three groups participated, two with evolution controls and one with the full feature set. The results showed that exploration controls were preferred to evolution, but the full feature set was most desirable.

The work of Gutierrez and Schrum (2020) took this idea a step further. They use a similar automatic GAN-based approach to the generation of individual dungeon rooms, as the previously described work, but combine this with designer-specified graphs describing the high-level dungeon layout for the game The Legend of Zelda. This work blends the abilities of GANs nicely to generate level segments, whose parameters can be controlled similarly to the two previous works, with an interface that allows a game designer to easily specify the desired high-level features.

#### 6.1.4 Fashion

Several works centered around fashion design, with solutions presented spanning across many different problems. The work of Kato et al. (2019) examined whether GANs can be used to generate novelty while preserving the inherent brand style. Their training set consisted of samples of a single brand design released over a 3-year span; this choice was made to maximize style consistency. The progressive growing of GANs (P-GAN) algorithm (Karras et al., 2017) was applied to produce a set of generated designs of varying resolutions outputted at three discrete training epochs. They then performed a user study and evaluation with professional pattern makers. The professionals were asked to evaluate the difficulty in creating a physical pattern from each design they were presented to evaluate the importance of both resolution and training time. Interestingly, neither factor had a significant impact. Of far greater importance was the professional’s experience with the underlying brand. Pattern makers are quite familiar with elaborating designs from rough sketches, but those designs are generally informed by brand design philosophy and principles. The authors noted that the professional’s impression of pattern-making difficulty was reduced when given rough dimensions and material suggestions and suggested that results could improve dramatically with much higher quality images.

Sbai et al. (2019) presented a system that generates novel fashion designs while allowing a fair degree of user control over several key features, importantly texture and color. The system builds on existing approaches to the generation of novelty by GANs (Elgammal et al., 2017) and facilitates a degree of fine control over the output and the degree of novelty introduced. In this way, the final tools are more useful to a potential designer than being presented with a set of novel designs. The authors performed some user studies to determine the degree to which their system produced preferred designs over state-of-the-art approaches and found a very significant improvement in likability scores for the garments produced by their system.

Cui et al. (2018) presented a tool for designers to visualize more complete fashion designs quickly. Users provide both an input sketch and a material. The system then applies the material to the sketch in an intelligent manner. In contrast to the previous work, the training data were broadly sourced, containing diverse designs, styles, and brands. The network architecture adapts BicycleGAN (Zhu et al., 2017b) by using fabric pattern samples to train the encoder so that only the material and color information are contained within the latent vector. A user’s sketch constrains the shape of the final generated image, and the color and material are constrained by the input pattern. The final sketch-based UI is straightforward to use, and one could imagine it being used in both recreational and professional settings. From a professional’s perspective, one could imagine the benefit of quickly visualizing different patterns on the same design, saving valuable production time. This work, unfortunately, omits a user study. A study may have yielded interesting findings as the visual fidelity of the produced designs is very impressive and among the best found among all papers reviewed. As we saw from the previously discussed paper, practitioners perceived difficulty in creating physical patterns from the generated designs were mitigated through material specification. Investigating how professionals perceived this tool with material specification and improved visual fidelity would be highly interesting.

**FIGURE 9:**
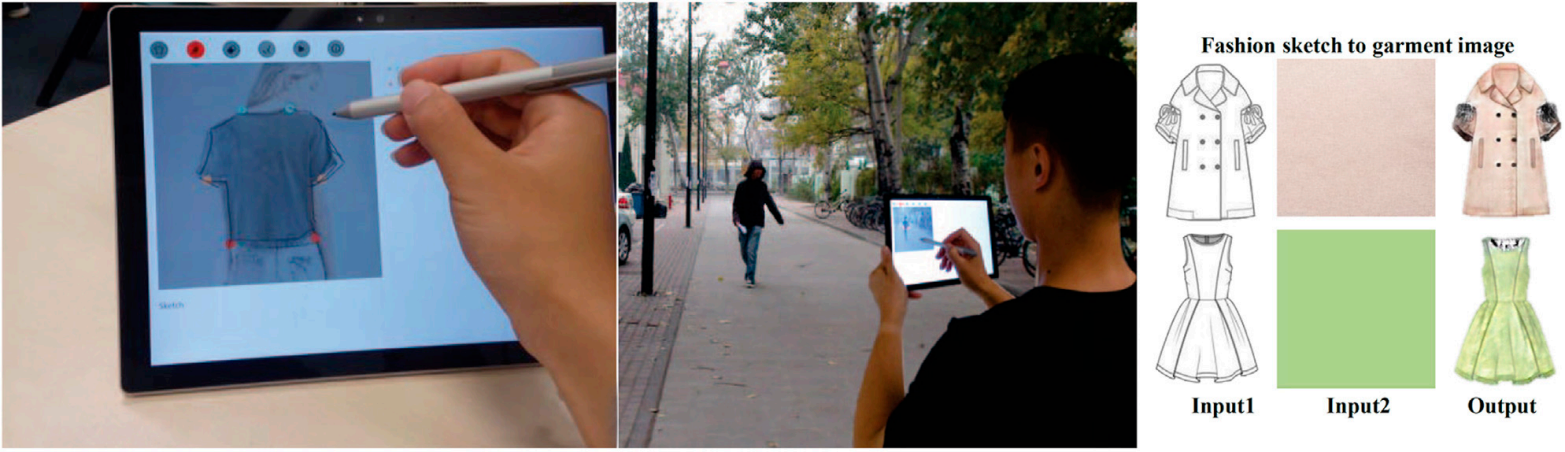
**Left:** The interface designed by Zhao and Ma (2018) in action, an AR system for *in situ* fashion design. **Right:** When designing, it can be cumbersome to iterate overall the possible material options. Cui et al. (2018) presented a system that attempts to optimize the process.

Zhao and Ma (2018) described an *in situ* augmented reality (AR)–enhanced fashion design system powered by AI. In this case, the authors detailed the thought process behind their design decisions. They consulted with design professionals in determining the feature set for their interface and considered their common practices and working habits. They determined that modern fashion designers often look to street style, taking inspiration from spontaneous urban fashion trends. The authors decided on an AR system to empower designers to sketch and design garments *in situ* quickly. One of the issues with generative networks is that they are bound to the data set upon which they are trained, but designers have different styles and techniques. To compensate for this and create a more generalized system, the authors decided on a two-step compensation method. The author first marks up the captured images with familiar landmarks (e.g., hemline and sleeve end). These landmarks are then used as a compensation signal for the second network to cater to sketch styles that lay outside of the trained network’s representation. While the system results lack the previous example’s visual quality, the interface and design processes are much improved (Figure 9: left). The authors kept the end-users in mind throughout, and the building blocks are in place for a viable, usable system. The AR component is a little limited where there is little essential difference between the desktop and AR in practice. However, a natural extension would be to use AR to dynamically map the generated designs to the model.

Cheng et al. (2020) introduced a novel approach for language-based interactive image editing. A database of image of fashion items alongside textual description was created and used in network training. During a session, a virtual agent takes natural-language directions from the user as the input. Based on the directions, the agent modifies the current image accordingly. In this way, the user can arrive at their desired design.

Dong et al. (2020) presented a system very similar to the method of Jo and Park (2019) (see section 6.1.5) but applied in the fashion space. The authors conditioned their network on data containing full-body models for better performance when working on clothing.

#### 6.1.5 Two-Dimensional Art

Considered a significant early work in the GAN space, the iGAN system developed by Zhu et al. (2016) was among the first systems that facilitated interactive feedback with a GAN-based model (it also heavily influenced the architecture around which many of the examples in this section are based). A user selects an image, which is projected into a low-dimensional latent representation using a GAN. The user then uses various brush tools to achieve the rough desired shape and color requirements visualized in the low-dimensional model in real time. At the final step, the same series of transformations are applied to the original image to generate a result. The system can also be used to design from scratch as even from a few simple strokes, the generator will do its best to generate a plausible result. The work of Chen et al. (2018) presents a similar painting interface to the previous example, with the intent this time to translate rough user sketches into more esthetically pleasing results. Their work builds on the VAE-GAN model (Larsen et al., 2016), generating far crisper images than merely using an AE model while maintaining many of their benefits.

**FIGURE 10:**
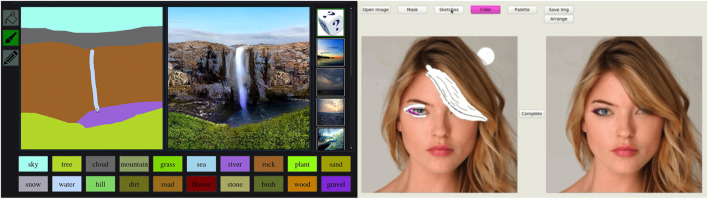
**Left:** The interface for the GauGAN system (Park et al., 2019). The user can create realistic landscapes with just a few simple input strokes. **Right:** Jo and Park (2019) developed a very familiar painting-based interface that allows users to alter photographs via sketches.

Park et al. (2019) presented a system, commonly known as GauGAN, capable of turning rough sketches into photorealistic pictures (Figure 10: left). Their system is built on top of the pix2pixHD (Wang et al., 2018) algorithm, introducing the SPADE (SPatially ADaptivE Normalization) normalization technique. Traditionally, normalization attempts to learn the affine layers after the normalization step, and so semantic information from the input tends to be “washed away.” SPADE learns the affine layer directly from the semantic segmentation map so that the input’s semantic information can be kept and will act across all layer outputs. Users provide the system with a sketch, in effect a semantic map, and a style image. The resulting image is generated in real time and highly responsive to user input. The SmartPaint system (Sun L. et al., 2019) presents a system and interface that closely mirrors the previous example. From an interface perspective, the main point of difference is that the system recommends a set of reference material from the dataset (representing the most similar examples to the user input) based on the user’s input sketch. In this way, the system attempts to guide the user toward more realistic, known examples while still allowing a large degree of creative flexibility.

There has been a considerable amount of recent research around image generation based on an input image and set of controllable parameters (Lee and Seok, 2017; Alqahtani et al., 2019). The recent work of Jo and Park (2019) builds upon this research and presents a sketch-based image-editing tool that allows users to alter images in a variety of ways (altering facial geometry, adding makeup, changing eye-color, adding jewelry, etc.). During an editing session, the user masks off, via sketch, areas of the image they want to edit and then sketch in the changes they want using their free-form artist license (Figure 10: right). They can also use color brushes to sketch features of that color (e.g., hair color). The system is highly performant, and the generated image adapts in real time to user input. The system also operates on relatively large images, 512 × 512 pixels, which increases real-world usability, as does the feel of its interface, which is much like any professional painting tool.

Ho et al. (2020) presented a novel sketch-based generation network for full-body images of people. The authors used semantic key points corresponding to essential human body parts as a prior for sketch-image synthesis. The authors demonstrate some impressive results, even given very course input sketches.

The art of inking is a refining process that builds on artists’ sketches. Inking refines the sketch, drawing emphasis on certain areas and lines of the sketch, and is a crucial tool in creating depth and perspective. Several image-editing suites^4^ provide automatic inking tools and features. Building upon their prior work, Simo-Serra et al. (2018) presented a system that uses GANs to transform a user sketch into an inked (a process the authors refer to as sketch-simplification) image. The network was trained jointly on both a supervised (a series of professionally drawn sketches and corresponding inked image pairs) and unsupervised (rough sketches and line drawings) datasets by employing an auxiliary discriminator network. By combining supervised and unsupervised data in this way, the system can more easily handle a wide variety of artist styles. The human–AI collaborative loop is not explored in depth, but the system does provide real-time feedback, and a user study could further validate the impressive results.

After inking, the next stage in developing a finished comic artwork is coloring. Ci et al. (2018), Hati et al. (2019), and Ren et al. (2020) all presented frameworks for user-guided line-art colorization. Automatic colorization of line art is a challenging task, as the colorization process must achieve a pleasing result while keeping the texture and shading of the original work. Line art, by its nature, does not provide any semantic information. To overcome this problem, the authors developed systems where the user sketches on the image providing information to the system about desired colors, location, etc. All articles used large datasets of colorized anime images, corresponding line art, and a pixel hint mask to train the network. One of the key improvements of the Hati et al. work was stroke simulation rather than simple pixel sampling to provide the color hints during training. Unfortunately, the works did not perform end-user evaluation studies, as the PaintsTorch system is one of the most feature-rich among the reviewed literature. Architecturally, the work presented by Zhang et al. (2017) is quite similar. Here, the authors trained their network on gray-scale photographs and their corresponding color images. The collaborative system allows users to add color landmarks and adjust them with real-time feedback to the gray image, and the system generates plausibly colorized images. The authors note that it is not always easy for users to select colors in an esthetically pleasing or realistic manner. To mitigate this problem, the system gives the user feedback about the colors they may wish to use, based on a predicted co-distribution, guiding them toward a good result. The authors did perform a user study. A group of nonexpert users was given 1 min to colorize a series of photographs, and the results were then passed through a real vs. fake Amazon Mechanical Turk (AMT) test.^5^ The automatic colorization performed reasonably well, but with user input, the number of images passing as real examples significantly increased and increased again when user color recommendations were used. While the user interface presented by Ren et al. (2020) is similar to the previous work, the internal architecture differs. An innovative two-stage interactive colorization based on superpixel color parsing was used to generate better results. The authors also proposed metrics for quantitative result evaluation.

All the design tasks that we have covered till now have been aimed at expert or semi-expert users. Zou et al. (2019) presented an example of human–AI collaboration primarily designed for children. The system is trained on a set of scene sketches and cartoon-style color images with text descriptions. The system allows users to progressively colorize an image, via simple natural language-based instructions. Users can refine the result interactively, specifying and colorizing specific foreground objects to match their requirements. An extensive series of validation experiments were run, looking at criteria such as performance and generalization. A more in-depth look at how children interacted with the system would be of real benefit, but we acknowledge the difficulty in performing such studies.

#### 6.1.6 3D Art

Normal maps are a commonly used tool in efficiently representing complex 3D shapes, adding depth and lighting to otherwise flat images. Su et al. (2018) presented a human–AI collaborative tool to generate normal maps from user sketches in real time. The authors used a slightly modified version of the popular pix2pix (Isola et al., 2016) algorithm and trained the network on a database of sketches with corresponding normal maps and a single-channel point-mask (user-defined hints). At runtime, the user can sketch and watch in real time as the normal map is generated. The user can select points on the image and manually adjust as needed (this point is adjusted in the point mask), allowing for fine-grain adjustment of the normals as needed. The final system is intuitive, with simple but responsive interactive controls, and the generated maps are of high quality, superior to those achieved by a selection of the other state-of-the-art algorithms. The authors conducted a small pilot study to look at perceptual loss for the rendered normal maps against several different methods, and their system performed significantly better than the other algorithms.

One of the most impressive systems reviewed was the terrain authoring tool presented by Guérin et al. (2017). The authors trained several GANs, or terrain synthesizers, corresponding to different sets of topological features. The training set was assembled through automatic conversion of example patches of the landscape into user-like sketches and contours. During terrain authoring, the artist provides a rough sketch. The sketch defines features such as rivers, ridges, some altitude cues, or a combination of them. The input is given to the sketch-to-terrain synthesizer that generates a plausible terrain from it in real time. If the result is not satisfactory, the user can re-edit the sketch and rerun the synthesis or remove parts of the terrain that will then be completed by the eraser synthesizer. After the coarse sketch is finished, the user can erode the terrain by running the erosion synthesizer. It should be noted that this level of performance and interactivity had not been seen before, even in professional tools. To evaluate and validate their system, the authors conducted a user study with both expert and nonexpert groups. After generating a landscape with specified features, the participants were asked to evaluate the system scale according to three criteria: (1) Does the generated terrain follow the sketch? (2) Is the system reactive? And finally, (3) is it easy to express one’s intent? The system scored very highly on all criteria.

The work of Zhao et al. (2019) zoned in on the task of adding high-fidelity detail to landscapes. Rather than outputting a landscape from a sketch, it amplified detail on an existing coarse landscape. A novel approach to embedding landscape “themes” into a vector space is described, giving artists and end-users control over the result’s look. The system is also performant enough for interactive edition, a crucial criterion for artists. The nature of the embedding space also allows for interpolating between them, allowing for exploration of themes outside the example space.

The system proposed by Liu et al. (2017) presents an interactive 3D modeling tool, assisting users in designing real-world shapes using a simple voxel-based interface. The system builds on the 3D-GAN model (Wu et al., 2016) by adding a projection operator that maps a user-defined 3D voxel input to a latent vector in the shape manifold of the generator that both maintains similarity to the input shape but also avoids areas of the latent space that generate unrealistic results. The training set consisted of a collection of shapes within a broad object category represented by voxel grids (the authors demonstrate results for planes, chairs, and tables). The method attempts to avoid “bad” or unrealistic areas of the latent space by training a projection model that attempts to balance similarity of the input to output with generating something very close to an existing sample. During a session, the user quickly builds up a simple voxel shape representing a rough approximation of the desired output in a voxel editor. Once finalized, the user hits the “SNAP” button, which triggers the generator and generates a result. In this way, the user interacts with the system to finalize their design. The system represents work in progress. The final output of a user session would need significant editing before being used in a production environment. However, the interaction between the AI and the user is intuitive and straightforward, and a similar approach may become more relevant as the quality of 3D-generated results improves.

The work of Wu et al. (2018) presented a very novel system to generate paint strokes using GANs. Traditionally, to generate realistic brush and natural media behavior (e.g., watercolors and oils), a fluid or physical simulation approach is adopted (Chen et al., 2015). Here, the authors replaced the paint simulation with a neural network (the brush strokes were still simulated). The model was trained on data generated by a physically based oil painting simulation engine (the inputs being corresponding height fields, color fields, and stroke information). During a live painting session, the network’s input consists of the existing paint on the canvas and the new stroke drawn by the user, and it outputs a predicted height map and color map of the new stroke. The system is highly interactive, and examples of some stunning user creations are presented. The system significantly outperforms their previous, simulation-based work and presents a new avenue for exploration with other natural media painting simulations such as watercolor or pastels.

#### 6.1.7 Sport

In BasketballGAN (Hsieh et al., 2019), the authors present a novel approach to human–AI collaborative play design. Basketball has a long history of coaches using clipboard sketches as a tool for play design and to convey those plays to their players. One need only turn on any high-level televised basketball game to see this in practice. One of the drawbacks of this design methodology is that it is static. It does not explicitly cater for how opposition players may react. Having an instinct for how the opposition will behave is purely down to the coach’s skill in understanding both the game and the skill sets of the opposing players. BasketballGAN gives the play designers the same primary tool they are used to and augments it with AI. The network was trained on a player movement dataset released by NBA. The system takes as input a sketch from the designer and outputs a dynamic play simulation (Figure 7: top right). To maintain the realism of the resulting simulations, several loss functions were described for dribbling, defending, passing, and player acceleration to guide the network. These heuristics prevent abnormal player behaviors on the court. The resulting system produces very plausible 2D simulations, and in this way, a coach can analyze their play designs and get an instant prediction on how the opposition may counter it. Using this information, they can iterate over the play to improve it or avoid passing to a player likely covered by a skillful defender, and so on. A small user study was performed to examine the plausibility of the generated results. Three groups, with varying levels of basketball knowledge, were asked to answer whether they thought a sample of generated and real plays was real or fake. Only the most expert group could distinguish the generated plays above the chance level, proving that the system could prove a viable real-world tool with further refinements.

### 6.2 Research Question 2: What Are the Limitations of Studies and Approaches in Generative Adversarial Networks-Enabled Human–Artificial Intelligence Collaborative Design Tools?

Given the early stages of human–AI collaboration research in the generative space, many of the articles reviewed presented effectively “toy” examples or case studies that were deliberately scoped to smaller examples to avoid the combinatory explosion problem. The applicability of some of the examples presented in this article will be tested as further research is conducted on more complex examples, but as the work stands now, very few of the systems described would be fit for a production environment.

Due to the complexity of AI and ML, from an algorithmic and architecture perspective, there is a gap in knowledge between interaction/user-experience designers and ML engineers when it comes to understanding ML’s limits, what it can and cannot achieve (Yang, 2018; Yang et al., 2020). Barring two examples (Guérin et al., 2017; Zhao and Ma, 2018), none of the other reviewed works discussed the gathering of end-user requirements, user experience design with any degree of detail. This may reflect the fact that ML engineers are driving the technology at this nascent stage. If AI technology is going to bridge the gap between algorithmic and human concerns, then HCI and UX designers have a vital role to play. From a research perspective, having a stronger initial focus on requirements gathering and human concerns would significantly improve both the final system and ground them in real-world practical problems.

Related to the previous point, the lack of systematic, robust user studies presents a significant limitation of the studies presented in this review. Close to half of the systems were not tested with users (*n* = 10), or when they did, little details of the testing were published (*n* = 6). Participant counts varied greatly, from 6 to 26. Some studies used experts, and some used novice users. Optimally, both groups’ performance would be examined during a study, but only two articles adopted this approach.

We are currently living in a 4K, soon to be 8K, world when considering consumers’ expected image resolution. Further to consumer displays, there has been increasing adoption of immersive environments with massive resolutions (Lock et al., 2018; Bourke and Bednarz, 2019). Due to the computational cost, architecture complexity, and difficulty in training GAN models, the current state of the art outputs images at 512 × 512 px. While some of these results are very impressive and have garnered much media attention, how much real-world value and penetration will these systems achieve without a marked increase in visual fidelity? There is no doubt that the quality of results will continue to improve as architectures evolve, but right now, it remains a major limiting factor.

### 6.3 Research Question 3: What Are the Future Research Avenues and Directions in the Domain of Generative Adversarial Networks-Enabled Human–Artificial Intelligence Collaborative Design Tools?

The “Double Diamond” design process model (UK, 2005) is among the most well-known and cited extant design process visualizations. It is referenced widely in the HCI/ML literature, and authors have attempted to adapt it to cater to ML systems (Yang et al., 2020). ML presents some fundamental problems that make its incorporation into such a design process challenging. First, rapid prototyping of ML systems can be very difficult to achieve in practice. Networks can take a long time to train and iterate over. Second, the results of the developed system are fundamentally constrained by the available data. Working with artists and designers to investigate how current processes can be adapted to cater to ML remains an essential avenue for research.

One fascinating piece of work was the BasketballGAN framework (Hsieh et al., 2019). It was notable that it was the only work that looked to visualize not simply a result, given a proposed design, but also a visualization of how that result would evolve over time. While this is not a genuinely novel concept, many simulation-based approaches exist to solve similar problems. It does represent a novel approach in the GAN space. Taking crowd simulation as an example, we see several GAN-based solutions for modeling behavior (Gupta et al., 2018; Amirian et al., 2019), but these models only take current agent states into account. A wide range of factors affect crowd behavior, for example, cultural factors (Fridman et al., 2013), density (Hughes et al., 2015), and group goals (Bruneau et al., 2014). Combining a similar approach to BasketballGAN, current methods could greatly improve crowd behavior and allow exploration of semi-scripted scenarios. Similarly, this concept could be extended to many problems and research fields.

One of the notable aspects of many of the works that we have reviewed is that users get instantaneous visual feedback based on their input (Keim et al., 2008). This allows the user to develop a relationship with the system and understand its features and limitations. As we mentioned in the previous section, ML solutions can fail in highly unpredictable ways. These failures can lead to a loss of user confidence and trust. One emerging field of research that seeks to mitigate this problem is XAI (Biran and Cotton, 2017; Hughes et al., 2020). XAI aims to look within the black-box and extract information or explanations for the algorithm’s output. In addition to providing tools to assist with trust and accountability, XAI can assist with debugging and bias in ML. The inputs and outputs and network design of ML algorithms are ultimately still decided with human input (human-in-the-loop) and, as such, are often subject to human errors or bias. Explanations from XAI-enabled algorithms may uncover potential flaws or issues with this design. Bau et al. (2019) presented DissectionGAN, a framework designed to examine the extent to which GANs learn image composition. To train their system, the authors first generated a series of images and then identified neurons within those images that correlated with meaningful object concepts. A user can switch these neurons on or off using their system, and the corresponding objects will be added or deleted. In this way, the system extracts meaning from the network that it can relay to the user in a useful manner. The LogicGAN system, presented by Graves et al. (2020), adapts recent advances in XAI (Lundberg and Lee, 2017) to the GAN space. Ordinarily, the discriminator network of a GAN simply reports one real-numbered value of corrective feedback to the generator network. LogicGAN incorporates an explanation network that allows for additional information regarding what features were important/unimportant in the discriminator’s decision back to the generator, in effect “explaining to the AI.” The explanations can also be explored by a user or ML engineer. These recent examples represent important steps forward in improving user trust and potential avenues for further research.

There is a fundamental question around a generative model’s ability to navigate outside its example space, generating more than simply re-combinations of the input. In section 6.1.4, we discussed some examples from the fashion field; one nonacademic example of note was the work by the cross-discipline team responsible for the Internet series of case studies, “How to generate (almost) anything” (Cameron and Yanardag, 2018). Their fashion design case study closely matched the work of Kato et al. (2019), but they trained their model on a database of cover art of vintage sewing patterns. Due to the restricted training time, the authors noted that the AI made some interesting mistakes, such as combining standard sleeves and bell sleeves within the same dress. Also, it tended to blend in elements from the background into the final design. These “mistakes” were inspirational for the pattern makers and led to final patterns that would probably not exist but for the training restrictions. In essence, this poses a critical question around the power of generative models. Often, the model is merely generating re-combinations of existing ideas. This is a limitation of an ideal GAN, since a perfectly trained GAN generator will reproduce the training distribution. Such a model cannot directly generate an image based on new fundamental principles because such an image would not look anything like it has seen in its training data. Other artists are explicitly exploring this idea (Olszewska, 2020), using GANs to create interesting new artworks. It may be the case that an imperfect GAN can be more artistically interesting than its ideal counterpart.

## 7 Limitations

Due to the criteria we imposed, the final sample size of articles is relatively small, limiting what broad conclusions or models can be elaborated at this point. However, it does represent the state of the research in the area at the moment. This would indicate that there is a large space for researchers to examine and exploit.

## 8 Conclusion

Leveraging the power of generative networks to create interfaces and systems that add to the creative toolbox of design practitioners is still in its early stages. This review has explored the current literature in human–AI collaboration involving GANs in the design space. We have shown that while the work in the area is still nascent, some powerful tools are starting to emerge. Trends are beginning to appear in terms of areas that researchers are focusing on, sketch-based interfaces, *in situ* design, and end-user–driven interface design.

This article has described current approaches, while also identifying a range of limitations in this field of research, primarily finding a lack of focus on the end-user when developing training sets and designing interfaces, and limited outcomes in terms of scalability or professional usability. If this technology is going to make the break-through to mainstream adoption, a stronger focus on collaboration and the end-user is needed.

## Footnotes

1
www.autodeskresearch.com/projects/dreamcatcher

2
www.sidefx.com/products/houdini

3
www.foundry.com/products/nuke

4
helpx.adobe.com/au/illustrator/using/image-trace

5
mturk.com
