# Supplementary material for: Generative Adversarial Networks–Enabled Human–Artificial Intelligence Collaborative Applications for Creative and Design Industries: A Systematic Review of Current Approaches and Trends
Source: Front Artif Intell. 2021 Apr 28;4:604234. doi: 10.3389/frai.2021.604234 (PMC8113684; doi:10.3389/frai.2021.604234)
Supplement: Supplementary file 1 [file Table1.DOCX]

Supplementary Material

# List of Papers Reviewed

| **Authors** | **Year** | **Title** | **Interface Type** | **User-Study** | **Domain** | **Pub Type** |
| --- | --- | --- | --- | --- | --- | --- |
| Zhu et al. | 2016 | Generative Visual Manipulation on the Natural Image Manifold | Sketch | Beautification | Art | Conference |
| Liu et al. | 2017 | Interactive 3D Modeling with a Generative Adversarial Network | Landmark | Beautification | Art | Conference |
| Zhang et al. | 2017 | Real-Time User-Guided Image Colorization with Learned Deep Priors | Landmark | Beautification | Art | Journal |
| Guerin et al. | 2017 | Interactive Example-Based Terrain Authoring with Conditional Generative Adversarial Networks | Sketch | Beautification | Art | Journal |
| Kelly et al. | 2018 | FrankenGAN: Guided Detail Synthesis for Building Mass Models Using Style-Synchonized GANs | Sketch; Landmark | Beautification | Architecture | Journal |
| Chen et al. | 2018 | Doodle Master: A Doodle Beautification System Based on Auto-Encoding Generative Adversarial Networks | Sketch | Beautification | Art | Conference |
| Wu et al. | 2018 | Brush Stroke Synthesis with a Generative Adversarial Network Driven by Physically Based Simulation | Sketch | Beautification | Art | Conference |
| Ci et al. | 2018 | User-Guided Deep Anime Line Art Colorization with Conditional Adversarial Networks | Sketch | Beautification | Art | Conference |
| Simo-Serra et al. | 2018 | Mastering Sketching: Adversarial Augmentation for Structured Prediction | Sketch | Beautification | Art | Journal |
| Su et al. | 2018 | Interactive Sketch-Based Normal Map Generation with Deep Neural Networks | Sketch | Beautification | Art | Journal |
| Zhao and Ma | 2018 | A Compensation Method of Two-Stage Image Generation for Human-AI Collaborated In-Situ Fashion Design in Augmented Reality Environment | Sketch; Landmark | Beautification | Fashion | Conference |
| Cui et al. | 2018 | FashionGAN: Display your fashion design using Conditional Generative Adversarial Nets | Sketch; Landmark | Beautification | Fashion | Journal |
| Volz et al. | 2018 | Evolving Mario Levels in the Latent Space of a Deep Convolutional Generative Adversarial Network | Parameter | Variation | Gaming | Conference |
| Hati et al. | 2019 | PaintsTorch: A User-Guided Anime Line Art Colorization Tool with Double Generator Conditional Adversarial Network | Landmark | Beautification | Art | Conference |
| Jo and Park | 2019 | SC-FEGAN: Face Editing Generative Adversarial Network With Users Sketch and Color | Sketch | Beautification | Art | Conference |
| Park et al. | 2019 | Semantic Image Synthesis With Spatially-Adaptive Normalization | Sketch | Beautification | Art | Conference |
| Zou et al. | 2019 | Language-Based Colorization of Scene Sketches | Language | Beautification | Art | Journal |
| Sun et al. | 2019 | SmartPaint: a co-creative drawing system based on generative adversarial networks | Sketch | Beautification | Art | Journal |
| Zhao et al. | 2019 | Multi-Theme Generative Adversarial Terrain Amplification | Sketch | Beautification | Art | Journal |
| Zheng et al. | 2019 | Content-Aware Generative Modeling of Graphic Design Layouts | Sketch | Beautification | Design | Journal |
| Hsieh et al. | 2019 | BasketballGAN: Generating Basketball Play Simulation Through Sketching | Sketch; Landmark | Prediction | Sport | Conference |
| Chen et al. | 2019 | An artificial intelligence based data-driven approach for design ideation | Landmark | Variation | Design | Conference |
| Sun et al. | 2019 | Adversarial Colorization of Icons Based on Contour and Color Conditions | Sketch; Landmark | Variation | Design | Conference |
| Zeng et al. | 2019 | Artificial Intelligence Augments Design Creativity: A Typeface Family Design Experiment | Landmark | Variation | Design | Journal |
| Sbai et al. | 2019 | DesIGN: Design Inspiration from Generative Networks | Landmark | Variation | Fashion | Conference |
| Kato et al. | 2019 | GANs-Based Clothes Design: Pattern Maker Is All You Need to Design Clothing | Sketch | Variation | Fashion | Conference |
| Noyman and Larson | 2020 | DeepScope: HCI Platform for Generative Cityscape Visualization | Landmark | Beautification | Architecture | Conference |
| Nauata et al. | 2020 | House-GAN: Relational Generative Adversarial Networks for Graph-constrained House Layout Generation | NodeBased | Beautification | Architecture | Journal |
| Ho et al. | 2020 | Sketch-guided Deep Portrait Generation | Sketch | Beautification | Art | Journal |
| Ren et al. | 2020 | Two-Stage Sketch Colorization With Color Parsing | Sketch; Landmark | Beautification | Art | Journal |
| Ho et al. | 2020 | Sequential Attention GAN for Interactive Image Editing | Language | Beautification | Fashion | Conference |
| Dong et al. | 2020 | Fashion Editing With Adversarial Parsing Learning | Sketch | Beautification | Fashion | Conference |
| Gutierrez and Schrum | 2020 | Generative Adversarial Network Rooms in Generative Graph Grammar Dungeons for The Legend of Zelda | NodeBased | Beautification | Gaming | Journal |
| Schrum et al. | 2020 | Interactive Evolution and Exploration within Latent Level-Design Space of Generative Adversarial Networks | Parameter | Variation | Gaming | Conference |
