# Supplementary material for: Generative Adversarial Networks–Enabled Human–Artificial Intelligence Collaborative Applications for Creative and Design Industries: A Systematic Review of Current Approaches and Trends
Source: Front Artif Intell. 2021 Apr 28;4:604234. doi: 10.3389/frai.2021.604234 (PMC8113684; doi:10.3389/frai.2021.604234)
Supplement: Supplementary file 2 [file DataSheet1.PDF]

Author	Year	Title	UX Modality	Operation	Domain	Type
Zhu et al.	2016	Generative Visual Manipulation on the Natural Image Manifold	Sketch	Beautification	Art	Conference
Liu et al.	2017	Interactive 3D Modeling with a Generative Adversarial Network	Landmark	Beautification	Art	Conference
Zhang et al.	2017	Real-Time User-Guided Image Colorization with Learned Deep Priors	Landmark	Beautification	Art	Journal
Guerin et al.	2017	Interactive Example-Based Terrain Authoring with Conditional Generative Adversarial Networks	Sketch	Beautification	Art	Journal
Kelly et al.	2018	FrankenGAN: Guided Detail Synthesis for Building Mass Models Using Style-Synchonized GANs	Sketch; Landmark	Beautification	Architecture	Journal
Chen et al.	2018	Doodle Master: A Doodle Beautification System Based on Auto-Encoding Generative Adversarial Networks	Sketch	Beautification	Art	Conference
Wu et al.	2018	Brush Stroke Synthesis with a Generative Adversarial Network Driven by Physically Based Simulation	Sketch	Beautification	Art	Conference
Ci et al.	2018	User-Guided Deep Anime Line Art Colorization with Conditional Adversarial Networks	Sketch	Beautification	Art	Conference
Simo-Serra et al.	2018	Mastering Sketching: Adversarial Augmentation for Structured Prediction	Sketch	Beautification	Art	Journal
Su et al.	2018	Interactive Sketch-Based Normal Map Generation with Deep Neural Networks	Sketch	Beautification	Art	Journal
Zhao and Ma	2018	A Compensation Method of Two-Stage Image Generation for Human-AI Collaborated In-Situ Fashion Design in Augmented Reality Environment	Sketch; Landmark	Beautification	Fashion	Conference
Cui et al.	2018	FashionGAN: Display your fashion design using Conditional Generative Adversarial Nets	Sketch; Landmark	Beautification	Fashion	Journal
Volz et al.	2018	Evolving Mario Levels in the Latent Space of a Deep Convolutional Generative Adversarial Network	Parameter	Variation	Gaming	Conference
Hati et al.	2019	PaintsTorch: A User-Guided Anime Line Art Colorization Tool with Double Generator Conditional Adversarial Network	Landmark	Beautification	Art	Conference
Jo and Park	2019	SC-FEGAN: Face Editing Generative Adversarial Network With Users Sketch and Color	Sketch	Beautification	Art	Conference
Park et al.	2019	Semantic Image Synthesis With Spatially-Adaptive Normalization	Sketch	Beautification	Art	Conference
Zou et al.	2019	Language-Based Colorization of Scene Sketches	Language	Beautification	Art	Journal
Sun et al.	2019	SmartPaint: a co-creative drawing system based on generative adversarial networks	Sketch	Beautification	Art	Journal
Zhao et al.	2019	Multi-Theme Generative Adversarial Terrain Amplification	Sketch	Beautification	Art	Journal
Zheng et al.	2019	Content-Aware Generative Modeling of Graphic Design Layouts	Sketch	Beautification	Design	Journal
Hsieh et al.	2019	BasketballGAN: Generating Basketball Play Simulation Through Sketching	Sketch; Landmark	Prediction	Sport	Conference
Chen et al.	2019	An artificial intelligence based data-driven approach for design ideation	Landmark	Variation	Design	Conference
Sun et al.	2019	Adversarial Colorization of Icons Based on Contour and Color Conditions	Sketch; Landmark	Variation	Design	Conference
Zeng et al.	2019	Artificial Intelligence Augments Design Creativity: A Typeface Family Design Experiment	Landmark	Variation	Design	Journal
Sbai et al.	2019	DesIGN: Design Inspiration from Generative Networks	Landmark	Variation	Fashion	Conference
Kato et al.	2019	GANs-Based Clothes Design: Pattern Maker Is All You Need to Design Clothing	Sketch	Variation	Fashion	Conference
Noyman and Larson	2020	DeepScope: HCI Platform for Generative Cityscape Visualization	Landmark	Beautification	Architecture	Conference
Nauata et al.	2020	House-GAN: Relational Generative Adversarial Networks for Graph-constrained House Layout Generation	NodeBased	Beautification	Architecture	Journal
Ho et al.	2020	Sketch-guided Deep Portrait Generation	Sketch	Beautification	Art	Journal
Ren et al.	2020	Two-Stage Sketch Colorization With Color Parsing	Sketch; Landmark	Beautification	Art	Journal
Ho et al.	2020	Sequential Attention GAN for Interactive Image Editing	Language	Beautification	Fashion	Conference
Dong et al.	2020	Fashion Editing With Adversarial Parsing Learning	Sketch	Beautification	Fashion	Conference
Gutierrez and Schrum	2020	Generative Adversarial Network Rooms in Generative Graph Grammar Dungeons for The Legend of Zelda	NodeBased	Beautification	Gaming	Journal
Schrum et al.	2020	Interactive Evolution and Exploration within Latent Level-Design Space of Generative Adversarial Networks	Parameter	Variation	Gaming	Conference
