# Supplementary figures and images for: Generative Adversarial Networks–Enabled Human–Artificial Intelligence Collaborative Applications for Creative and Design Industries: A Systematic Review of Current Approaches and Trends
Source: Front Artif Intell. 2021 Apr 28;4:604234. doi: 10.3389/frai.2021.604234 (PMC8113684; doi:10.3389/frai.2021.604234)

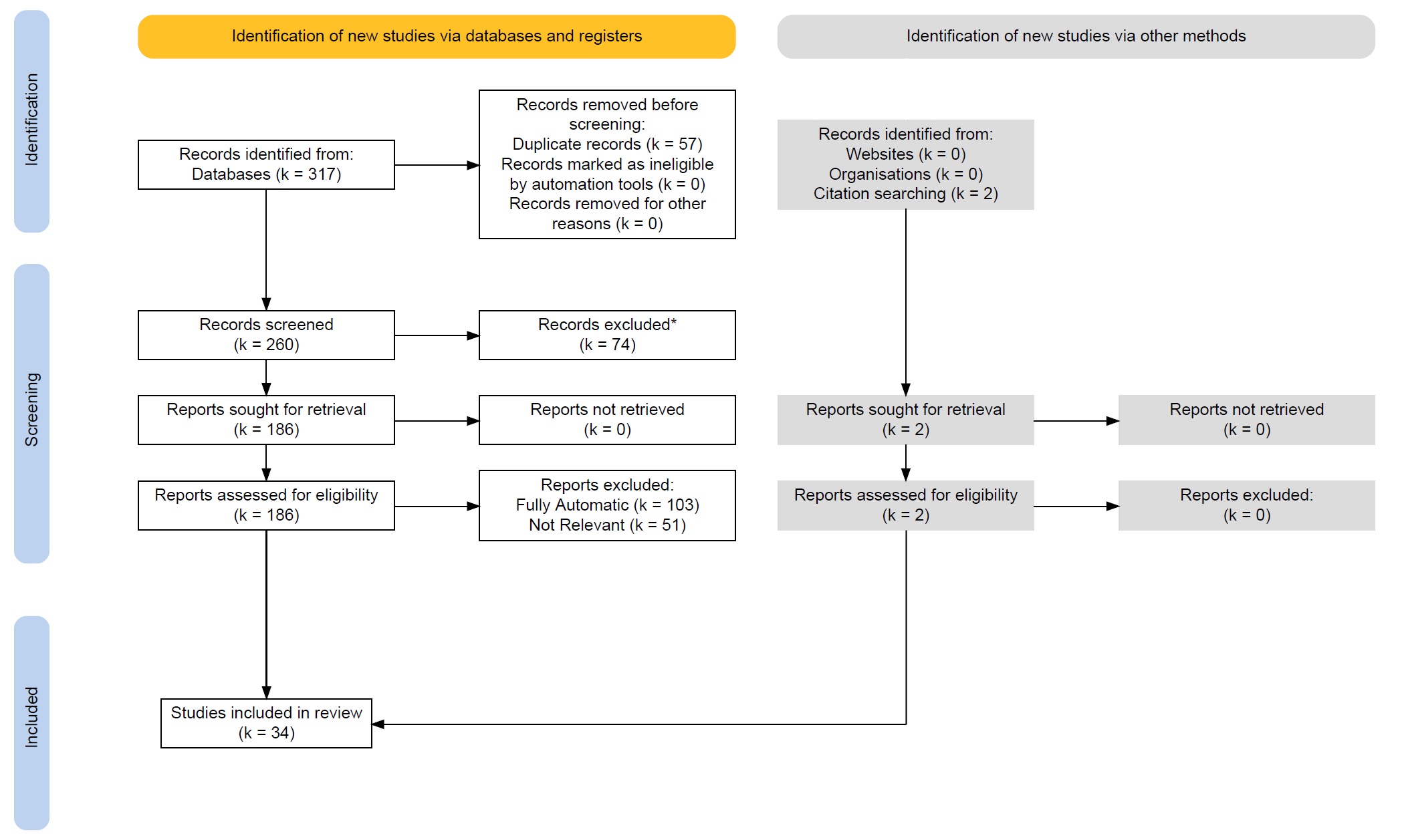

Supplement: Supplementary file 3 [file Table2.DOC]
